# Postharvest Operations of Cannabis and Their Effect on Cannabinoid Content: A Review

**DOI:** 10.3390/bioengineering9080364

**Published:** 2022-08-03

**Authors:** Pabitra Chandra Das, Alec Roger Vista, Lope G. Tabil, Oon-Doo Baik

**Affiliations:** Department of Chemical and Biological Engineering, University of Saskatchewan, 57 Campus Drive, Saskatoon, SK S7N 5A9, Canada; pcd476@usask.ca (P.C.D.); ajv715@usask.ca (A.R.V.)

**Keywords:** cannabis, cannabinoids, postharvest, drying, curing, storage, biosynthesis, pre-treatments, sorption isotherm

## Abstract

In recent years, cannabis (*Cannabis sativa* L.) has been legalized by many countries for production, processing, and use considering its tremendous medical and industrial applications. Cannabis contains more than a hundred biomolecules (cannabinoids) which have the potentiality to cure different chronic diseases. After harvesting, cannabis undergoes different postharvest operations including drying, curing, storage, etc. Presently, the cannabis industry relies on different traditional postharvest operations, which may result in an inconsistent quality of products. In this review, we aimed to describe the biosynthesis process of major cannabinoids, postharvest operations used by the cannabis industry, and the consequences of postharvest operations on the cannabinoid profile. As drying is the most important post-harvest operation of cannabis, the attributes associated with drying (water activity, equilibrium moisture content, sorption isotherms, etc.) and the significance of novel pre-treatments (microwave heating, cold plasma, ultrasound, pulse electric, irradiation, etc.) for improvement of the process are thoroughly discussed. Additionally, other operations, such as trimming, curing, packaging and storage, are discussed, and the effect of the different postharvest operations on the cannabinoid yield is summarized. A critical investigation of the factors involved in each postharvest operation is indeed key for obtaining quality products and for the sustainable development of the cannabis industry.

## 1. Introduction

**Figure 1:**
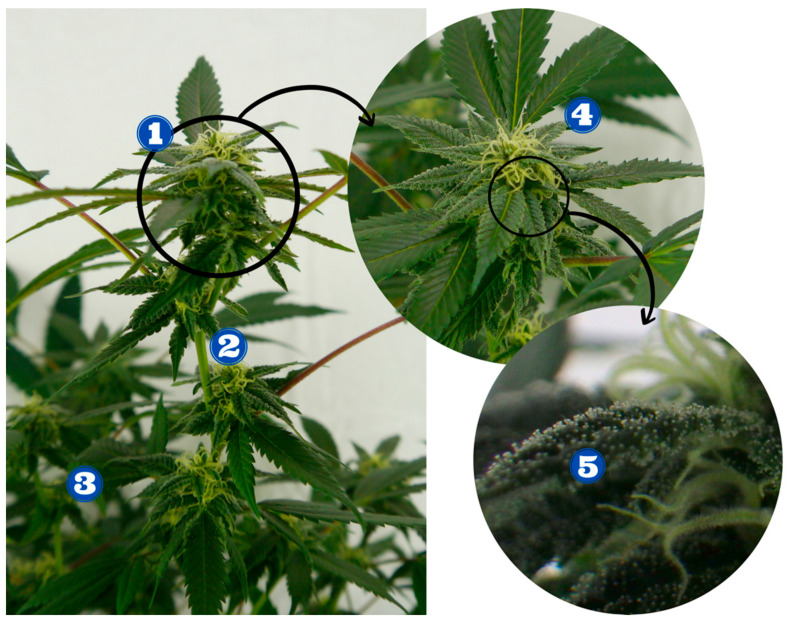
A cannabis plant: (**1**) mature bud/calyx/inflorescence, (**2**) stem, (**3**) fan leaves, (**4**) sugar leaves, and (**5**) trichomes (images were taken by M. Neufeldt and are used with permission).

Cannabis, scientifically known as *Cannabis sativa* L. from the family Cannabaceae, has been cultivated for ages and utilized as a potential source of fiber, oil, protein, and popularly, psychoactive and medicinal purposes [1]. Historically, cannabis products have been illicitly grown for recreational and social activities to appreciate their euphoric effects [2]. Cannabis contains more than 120 active compounds, which are classified to belong in a secondary metabolite group known as phytocannabinoids or cannabinoids [3] of which cannabidiol (CBD), ∆9-tetrahydrocannabinol (THC), cannabigerol (CBG), and cannabinol (CBN) are the most studied [4]. Terpenes, which are secondary metabolites usually associated with the flavor and smell profile of biological materials have recently been found to be relevant in terms of the chemical make-up of cannabis [5]. *Cannabis sativa* L. is often classified into three subspecies depending on physical or chemical characteristics, namely *Cannabis sativa* ssp. *sativa*, *Cannabis sativa* ssp. *indica*, *Cannabis sativa* ssp. *ruderalis* [6]. The cannabis plant (Figure 1) is holistically utilized to undergo different treatments after harvest for specific uses.

As shown in Figure 1, the cola or inflorescence of cannabis contains the most abundant amount of trichomes. Trichomes are glandular membranes containing secondary metabolites, phytocannabinoids, and terpenes, and are found to be most abundant on the surface of the cannabis inflorescence.

Extracts or products of cannabis have been studied for the treatment of different illnesses including neurological and psychiatric disorders [2], prevention of vomiting and nausea of cancer patients, boosting of hunger in acquired immunodeficiency syndrome (AIDS) patients [7], and the treatment of muscle spasms, spasticity and neuropathic pain, and chronic pain [8]. On the other hand, hemp has been produced for textiles, biocomposites, papermaking, biofuel, functional foods, and cosmetics [6]. Hemp seeds have been used in the processing of different edible foods like cookies, chocolate, peanut-cannabis butter blends, coffee, etc. The benefits outweigh the detriments brought about by cannabis as it has been legalized by more than 50 countries and 12 states of the USA for production, processing, and use [9]. Today, the cannabis industry is developing around the world, resulting in economic gain. The total cannabis market was anticipated at $3.5 billion in 2019, and a consistent increase up to $20.2 billion is expected by 2025. In Canada, production and marketing of cannabis have been legalized since October 2018 for medicinal and recreational purposes, although special monitoring and licensing from Health Canada is required in many cases. Total cannabis sales in Canada were around $210 million from mid-October to December 2018 and this value can reach multi-billions by 2021 [10]. This growing business sector should be given special focus to ensure safety and quality for the consumers.

Aside from positive effects, cannabis has been shown to cause detrimental health impacts in humans. Continuous usage of cannabis has been found to cause a variety of immediate and long-term detrimental consequences, including cardiovascular and respiratory problems, behavioral impairment, psychosis, schizophrenia, and mental illnesses [2]. People with cannabis use disorder have problems such as extreme mood shifts and memory-loss-related and concentration issues [11].

After harvest, different treatments are involved in the processing of cannabis-based food or non-food products. Drying is the most important postharvest operation involved in cannabis along with storage and curing. However, the present industrial drying involves a hang-drying method, which is inefficient and can cause an inconsistent quality of products. Thus, it is urgent to identify suitable drying methods that can enhance consistency in terms of quality for large-scale operations. In this paper, the postharvest treatments involved in cannabis processing and preservation are critically discussed and interpreted. In a broader context, this paper includes the following sections: phytocannabinoids and their decarboxylation; the postharvest operations of cannabis, including trimming, drying of cannabis (and the technical factors associated with these operations, such as equilibrium moisture content and sorption isotherms), curing, and storage and packaging; and finally, the effects of different postharvest treatments on the cannabinoid profile of cannabis. The relevant published articles were collected using the search engines Google Scholar, Scopus, and the library website of the University of Saskatchewan by inputting the keywords “cannabis, postharvest, cannabinoids” and the published articles or books from 2011 to date were considered. However, for specific interests like biosynthesis, sorption, pre-treatments, etc., the keywords were changed, and a specific publication period was not considered for extracting basic information.

## 2. Phytocannabinoids and Decarboxylation

Because it has various natural ingredients, cannabis is a chemically complicated plant. It includes secondary metabolites or phytochemicals called cannabinoids, a special type of terpenophenolic molecule [12]. Apart from cannabis, few other plants, such as cinnamon, clove, oregano, cocoa, black truffles, and black pepper, among others, also have cannabinoids in minor amounts. There are currently more than 120 cannabinoids found in the genus Cannabis that have different properties, structures, and uses [13]. THC and CBD are the most popular.

Radwan et al. [12] grouped cannabinoid compounds into 11 subcategories: Δ9-THC type (23 compounds), Δ8-THC (5 compounds), CBG (16 compounds), cannabichromene (CBC, 9 compounds), CBD (7 compounds), cannabinodiol (CBND, 2 compounds), cannabielsoin (CBE, 5 compounds), cannabicyclol (CBL, 3 compounds), cannabinol (CBN, 11 compounds), and cannabitriol (CBT, 9 compounds), along with miscellaneous type cannabinoids (30 compounds). According to Addo et al. [4], Δ9-THC, CBD, CBG, and CBN are the major cannabinoid groups because of their distinct nature and application.

**Figure 2:**
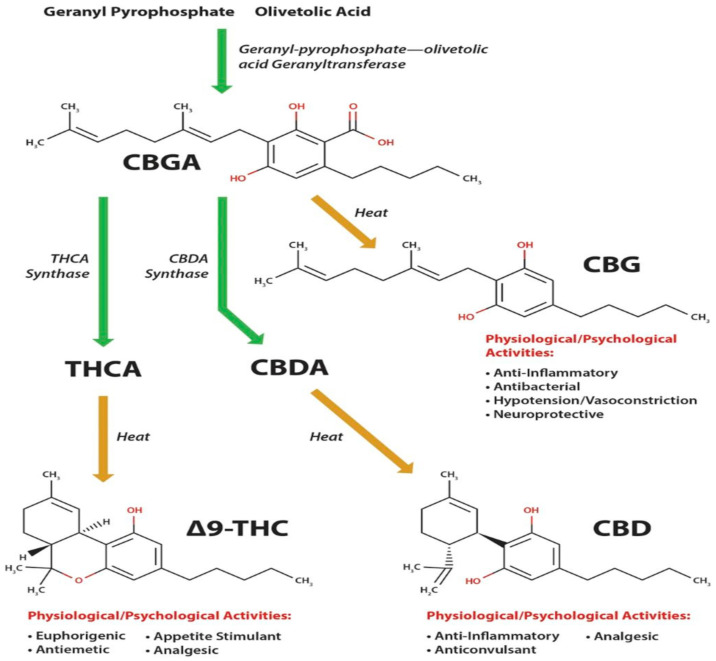
Biosynthesis of cannabinoids (adapted from Nachnani et al. [15] with permission).

The biosynthesis process describes the formation of the neutral forms of major cannabinoid compounds from their acidic form(s). In the biosynthesis process (Figure 2), cannabigerolic acid (CBGA) is produced by combining the primary molecules olivetolic acid and geranyl-pyrophosphate (CBGA) with the action of geranyl-pyrophosphate-olivetolic acid geranyltransferase enzymes, which is then used to synthesize cannabinoids [14,15]. Oxidocyclase enzymes convert CBGA into acidic forms of THCA, CBDA, and CBCA [4].

All enzymatically generated cannabinoids (including CBG) start off at an acidic state, that is then decarboxylated through heating to yield the functional or active form. With the application of heat, THCA, CBDA, and CBGA change into their active forms of THC, CBD, and CBG, respectively. Active cannabinoids have several physiological or psychological functions that could be either beneficial or detrimental. THC is euphorigenic, hunger-triggering, pain-relieving, and works against vomiting and nausea. CBD is not euphorigenic but works as an anti-inflammatory and anticonvulsant agent. On the other hand, CBG has antibacterial properties as well as working against neurologic disorders and inflammatory bowel disease [15].

A process called decarboxylation, which is the removal of a carboxyl group in the presence of heat, is required to convert acidic cannabinoids to their pure form. Thus, decarboxylation can be defined as the chemical reaction that removes a carboxyl (COOH) group from THCA and CBDA, resulting in THC and CBD, respectively. This reaction is facilitated by heating and vaporization. Wang et al. [13] reported that the decarboxylation of THCA to THC is more pronounced and very abrupt in a period of 60 min for temperatures greater than 100 °C. The lower temperature limit used for this study was 80 °C and it was reported that the concentration of THCA is still decreasing, following an almost linear decreasing trend in a period of 1 h. CBDA also follows a similar pattern. Recalling the spontaneous deterioration of THC to CBN, which is a less psychoactive cannabinoid [16], a holistic approach is usually taken when drying cannabis; thus, the use of a low drying temperature is practiced.

Entourage effect refers to the joint effects of taking these cannabinoids together with other minor cannabinoids and phytochemicals called terpenes (volatile aromatic compounds) being more beneficial than just taking pure THC or pure CBD [17,18]. Trichomes (as shown in Figure 1), which are the microscopic glandular membranes found on the surface of the cannabis inflorescence, contain cannabinoids and terpenes that are vital for psychoactive and medicinal effects. The glands enclosing these cannabinoids and terpenes allow them to be protected from external environmental factors that could hasten degradation [8]. Trichomes, not the buds, contain the cannabinoids that are vital for psychoactive and medicinal use; the temperature sensitivity of the cannabinoid- and terpene-containing trichomes is of prime importance when considering the postharvest unit operation conditions [19,20,21]. Terpenes have also been closely investigated as a possible solution to the taxonomic classification challenges that arise with the rampant genetic modification of cannabis cultivars to produce more secondary metabolites, focusing on high THC or CBD content in the illicit market in the years that cannabis was illegal. The chemical classification, or more specifically, the metabolomics of cannabis terpenes, is in the forefront of this emerging solution [18,22].

The stability data of these cannabinoids and phytochemicals during harvesting, drying, and storage are important assets for the emerging industry. According to Kader et al. [23], there are biological and environmental factors that influence the deterioration of biological commodities. Biological factors include: (a) respiration, which is the breaking down of biological components, such as carbohydrates, proteins, and fats into simpler substances; (b) the production of ethylene, which encourages senescence or degradation; (c) changes in the composition (in the case of cannabis), which involves the spontaneous deterioration of THC to CBN; (d) further growth and development, which may do more harm than good to plant quality; (e) water loss or transpiration, although this could be beneficial for cannabis; (f) pathological breakdown due to the presence of moisture; (g) physiological breakdown; and h) physical damage. The physical factors that encourage deterioration are temperature, light, relative humidity (RH), the presence of ethylene, environmental conditions, and the addition of other chemicals, such as fertilizers and fungicides [16,23].

## 3. Postharvest Operations Involved in Cannabis

**Figure 3:**
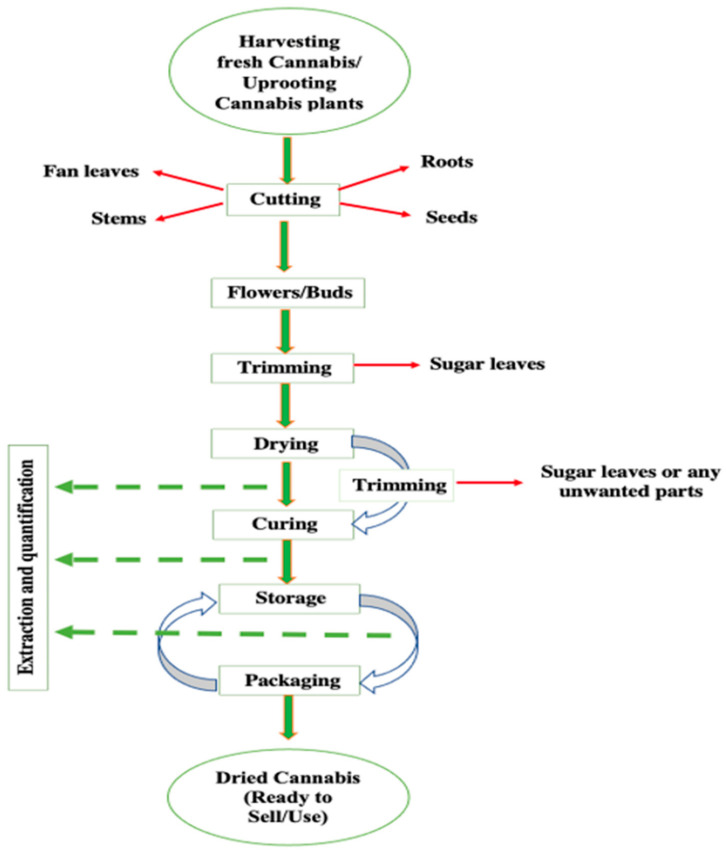
Postharvest operations involved in cannabis processing.

Maturity and harvesting of cannabis flowers depend on a number of factors similar to other plants, including day length, season, variety, soil fertility, temperature, etc. Generally, it takes six to sixteen weeks to harvest the necessary parts from cannabis plants [24]. The general operations involved in the processing of cannabis starting from harvesting and ending at marketing are depicted in Figure 3.

### 3.1. Trimming

During harvesting, different portions of cannabis are separated from the main plant and further processing is performed based on the target product types. The stems are used for the production of fiber, textiles, or animal bedding while the seeds are used for the extraction of oil, protein, and the processing of different food products. The cannabinoid-containing parts, the inflorescence along with stems, are removed from the main plant for pharmacological or recreational use. Stems and long fan or sugar leaves are cut to detach the inflorescence. This also helps in increasing the surface area for drying. Dried cannabis sometimes undergoes the trimming process to discard the less desirable parts, such as the sugar leaves, which have less cannabinoid content compared to the inflorescence. Size reduction processes in the food and agriculture industry often involve the use of mechanical force and the large material to separate physical parts or to divide larger particles into smaller more manageable ones. Size reduction processes are often classified based on the nature and direction of force applied to the process, such as compression, shear, or impact [25].

Trimming in the cannabis industry is a niche size reduction operation of removing the sugar leaves that cover the inflorescence, especially during drying. There are two iterations: trimming before the extensive drying process (wet trimming) or trimming after drying and/or curing process (dry trimming). An effective trimming process is delicate enough to preserve the structure of trichomes that house major cannabinoids, but effective enough to remove most of the sugar leaves [26]. Size reduction operations can be done before or after drying, depending on the producer’s preference and discretion and ease of flow of operations.

Current trimmer designs commonly use shearing techniques to reduce the amount of sugar leaves on the inflorescence. The mechanism for trimming uses a rotating cylinder with perforated walls or a set of pressurized wires that are designed to shear off the sugar leaves but not cut or slice through the inflorescence. The rotating cylinder is then inclined downwards to allow continuous flow with options for manual feeding and output collection or extra accessories for conveying the product into and from the trimming operation [27]. The perforated cylinders are then surrounded by a closed shell attached to a vacuum to collect the discarded sugar leaves, where ambient air drying may occur [27]. Improvements in the equipment design and final output quality in trimming operations are critical for better industry standards. These improvements should focus on the effectiveness of the process of sugar leaf removal with minimal damage and abrasion to the trichomes on the surface of the inflorescence.

### 3.2. Drying of Cannabis

The main postharvest operation of cannabis processing is drying. As heat is the main agent for the decarboxylation of cannabinoids, the appropriate drying temperature and conditions are very important to obtain good quality product with a maintained amount of THC, CBD, or other cannabinoids. The inflorescence of raw cannabis contains around 78 to 80% moisture, which needs to be reduced for safe storage and production of ready-to-use dry products [28,29]. Researchers are trying to identify suitable drying technology, which can replace the existing hang-drying practice. In this section, insights on drying and the current industrial practices with their pros and cons are discussed. Additionally, equilibrium moisture content, sorption isotherm, sorption isotherm models, and possible pre-treatments for the improvement of drying are also discussed.

#### 3.2.1. Mechanism of Drying

Drying is a popular unit operation usually characterized by the presence of a solid-liquid separation that is usually facilitated by the presence of heat, resulting in the evaporation of a liquid, which is water in the majority of cases. The definition of drying could also extend to solvent evaporation, dehydration of various biological materials such as in food and feed, the dehydration of salts, and the removal of hydroxyl groups from organic material [30,31,32].

Industrial drying involves the use of large amounts of energy usually in the form of heat, which can use different mechanisms, such as convection, conduction, radiation, or a combination of these. Certain amounts of sensible and latent heat need to be provided around the entirety of the material to achieve evaporation. Convection is characterized by a carrier gas or feed gas that is usually air, supplying the heat for evaporation of water or solvent. Conduction is the heat transfer mechanism during contact drying. The carrier gas in this context serves as the medium to which the solvent is evaporated. The radiation mechanism has different subtypes; penetrative radiation drying would consist of dielectric drying, such as radiofrequency or microwave drying, where heat is generated in the material rather than the heat diffusing into the material, which is non-penetrative [31,33].

Drying rate is the rate of moisture removal during a given drying process, obtained by the ratio of moisture removed to the time of drying. Total drying time is split into two major periods: (i) a constant rate period and (ii) one or more falling rate period(s). The constant rate period occurs when water is removed from the surface by evaporation and internal water movement is sufficient to keep the surface saturated. The surface temperature remains constant and is lesser than the circumambient drying air because the energy input rate is the same as the heat energy lost due to evaporation. In the falling rate period, the surface of a material is unsaturated, and the movement of interior moisture is lower than the rate of surface evaporation. During the first falling rate period, the drying rate is observed to fall as water content reduces due to increased resistance to evaporation, as well as a reduction in heat flowing into the material when the surface temperature rises to the heating medium temperature. However, the temperature of the material does not significantly increase or differ from the wet-bulb temperature. The second falling rate period commences when the partial pressure of moisture all throughout the material is lower than saturation. Heat flux from the hot air to the sample is very low as there is a small temperature gradient [32,33].

#### 3.2.2. Current Industrial Drying Practices for Cannabis

**Figure 4:**
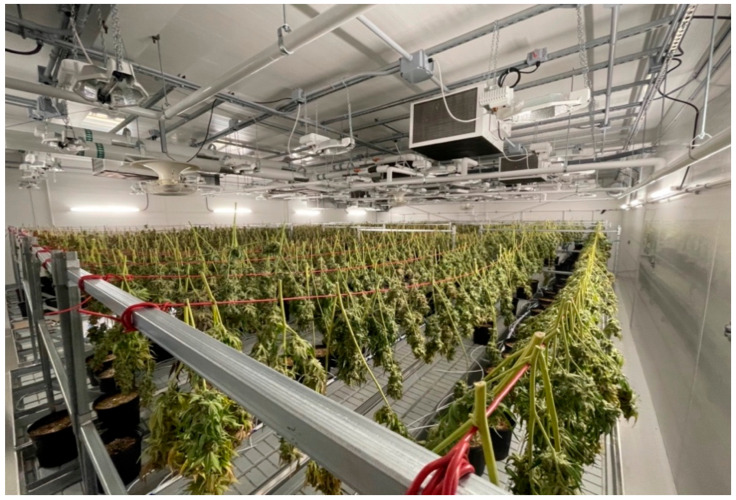
Hang-drying practice commonly used in large scale cannabis operations. Image taken by A. Vista (co-author).

Cannabis drying operations through the years have not evolved too far from the slow-drying method or low-temperature drying (Figure 4) that usually takes 5–6 d at near ambient conditions. The commonly used drying temperature is 18–21 °C and relative humidity is 50–55%. Currently, no model has yet been established for the prediction of the drying endpoint and total drying time. Postharvest processes are currently considered more of an art than a science as there are no standards for which the endpoint of drying is defined, and practices are based on word of mouth and are usually still subject to deviation. In the slow drying technique, cannabis stalks are hung on wire liners with the top buds or the cola upside down in isothermal room conditions until desired water activity is achieved. Screen drying is a variation of the slow drying process wherein the trimmed buds are dried in an enclosed and properly ventilated room by placing them on trays or screens. In this screen drying method, drying conditions are almost the same as in slow drying processes, but this process takes around 4 to 5 d to dry the cannabis completely [10].

Present industrial drying practices have several drawbacks such as a long processing time, chances of contamination by molds or fungi, and inconsistent quality in the final products. Researchers are trying to introduce modern and novel technologies in this sector. According to Chen et al. [34], drying conditions and methods dominate the drying time and quality characteristics of cannabis. Air drying, microwave-assisted convective drying, freeze drying, vacuum drying, microwave assisted freeze drying, intermittent (non-isothermal) drying, conveyor drying, radio frequency, and electrohydrodynamic drying, among others are the suggested modern drying techniques for cannabis [4,10,35]. However, critical identification of psychrometric behavior for devolvement of new drying technologies is equally important.

Psychrometric terms predominantly govern postharvest operations in the cannabis industry. Psychrometrics is the study of atmospheric air and its thermodynamic properties, typically focusing on psychrometric terms or parameters [36,37,38]. The interaction and control of these parameters are vital for success in an optimum quality-controlled process, storage of products, and packaging. In the cannabis industry, four humidity terms are used to describe the drying convective air: (a) vapor pressure, (b) relative humidity, (c) humidity ratio, and (d) water activity. These terms are usually common in psychrometric calculations together with dry and wet bulb temperature, enthalpy, dew point temperature, and specific volume [37]. However, relationships among the psychrometric parameters should be investigated as it would be helpful for cannabis drying and storage in different environmental conditions.

#### 3.2.3. Equilibrium Moisture and Sorption Isotherms

Equilibrium moisture content (EMC) is the amount of moisture in a biological material when equilibrium occurs. The corresponding relative humidity is the equilibrium relative humidity (ERH). The plot of product moisture versus air relative humidity at a constant temperature is called the equilibrium moisture content curve [32].

**Figure 5:**
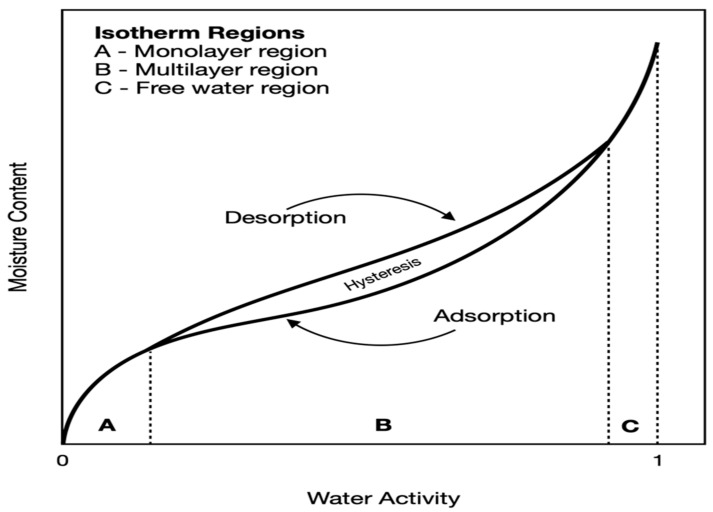
Sorption isotherms in biological materials (reproduced from [32,39,41]).

The EMC sorption curve appears in a sigmoidal shape due to how moisture is bound in a material. At low relative humidity values, shown in Figure 5 (region A), moisture is assumed to be bound tightly to the biological material’s surface. In this region, water exhibits a monolayer behavior where the amount of tightly held water is approximately like when a single water molecule is being held on each hydrophilic site across the surface of a biological material. The Langmuir isotherm, which is obtained by the monomolecular adsorption of vapor by porous particles in a finite volume of voids, can be used to approximate these areas [39,40].

The intermediate region (region B in Figure 5) is where water is more accurately described as “multimolecular” because water molecules are bound to different sites at different distances from the center of the biological material, and thus is easily pictured as multilayers of moisture across the biological material. The point of transition between the monolayer and multilayer occurs at relative humidity values of around 5 to 20%. The third region (region C in Figure 5) is the free water region, usually associated with water activity values greater than 0.9, where properties exhibited by dilute solutions are observed and water is loosely attached to the binding sites and is readily available for reaction to other substances.

Hysteresis is a phenomenon related to the means on which water is distributed across the biological material, and it explains the nature of biological materials to undergo structural rearrangements that affect the availability of hydrophilic sites to bind to water at re-adsorption as a result of the presence of an offset, as seen in Figure 5 [32,39].

Moisture activities in biological materials for drying processes are usually predicted from standard water activity thresholds to prevent the proliferation of unwanted biological contaminants. The United States Food and Drug Administration (FDA) defined water activity as the ratio between the vapor pressure of the substance itself, when in a completely undisturbed balance or equilibrium with the circumambient air media, and the vapor pressure of distilled water under similar conditions. This means that at different temperatures, the water activity of a substance may vary [42]. The FDA also defined water activity as equivalent to the equilibrium relative humidity:

(1) $$a_{w}=\frac{\mathrm{ERH}}{100}$$

where ERH is the equilibrium relative humidity (%).

Sorption isotherms are equilibrium moisture content and equilibrium relative humidity values at a constant temperature and pressure. The knowledge of the stability of water in a biological material such as cannabis is important in designing and optimizing drying operations, designing packaging materials, predicting quality and stability, and estimating the loss of moisture during storage [39,43].

##### Sorption Isotherm Models

To predict EMC based on the measurements of ERH, isotherm equations are generated based on standardized equations. ASABE Standards ASAE D245.7 [44] summarized five isotherm equations.

- Modified Henderson equation:

(2) $$\mathrm{ERH}\;=1\;–\exp\left[-A\;\times \left(T\;+\;C\right)\times {\left({\mathrm{MC}}_{D}\right)}^{B}\right]$$

- Modified Chung–Pfost equation:

(3) $$\mathrm{ERH}\;=1\;–\exp\left[-\frac{A}{T+C}\exp\left(-B\times {\mathrm{MC}}_{D}\right)\right]$$

- Modified Halsey equation:

(4) $$\mathrm{ERH}=\exp\left[-\frac{\exp\left(A+B\times T\right)}{{\left({\mathrm{MC}}_{D}\right)}^{C}}\right]$$

- Modified Oswin equation:

(5) $$\mathrm{ERH}={\left[{\left(\frac{A+B\times T}{{\mathrm{MC}}_{D}}\right)}^{C}+1\right]}^{-1}$$

- Guggenheim–Anderson–de Boer (GAB) equation:
  (6) $$\mathrm{ERH}=\frac{A\times B\times C\times \mathrm{ERH}}{\left(1-B\times \mathrm{ERH}\right)\times \left(1-B\times \mathrm{ERH}+B\times C\times \mathrm{ERH}\right)}$$
  where ERH = equilibrium relative humidity (decimal value); T = temperature (°C); MC_D_ = dry-basis moisture content (decimal value); and A, B, and C = equation constants, and the related data specifications based on which these constants were obtained are summarized in Table 2 of ASABE Standards ASAE D245.7 [44].

#### 3.2.4. Possible Pre-Treatments for Improvement of Drying of Cannabis

Pre-treatments are the physical or chemical treatments used prior to any process to enhance the quality of the product as well as to improve the process. Drying pre-treatments can reduce the drying time as well as the energy consumption during the drying process [45]. A number of physical and chemical pre-treatments are frequently used for the drying of foods, medical plants, herbs, spices, etc. Physical pre-treatments include steaming, blanching, microwave heating, ohmic heating, ultrasonic treatment, pulsed electric field, high pressure processing, freezing, etc., while chemical pre-treatments are conducted by treating the samples with different chemicals, such as sulfur dioxide, carbon dioxide, ozone, alkali solution, acid solution, etc. [45]. Do these pre-treatments improve the cannabinoid profile of cannabis? Up to now, there is no clear answer and very little research is conducted so far on the analysis of the potential of pre-treatments for maintaining the level of cannabinoids and terpenes. However, these pre-treatments can reduce the drying time. As decarboxylation requires heat treatment for the conversion of cannabinoids from their acidic form into their neutral form, thermal or physical pre-treatments could accelerate the process. This section discusses some possible pre-treatments that can be applied in the cannabis industry.

##### Microwave Heating

Electromagnetic waves with wavelengths ranging from 0.001 to 1 m and frequencies ranging from 300 to 300,000 MHz are called microwaves [46]. The frequency of electromagnetic energy equaling 915 MHz is used in the industry due to better penetration depth. During MW heating, heat is generated throughout the volume simultaneously due to the absorption of electromagnetic waves of certain frequencies in the form of energy [47]. Microwave heating helps in the improvement of operational speed, product quality, and energy use efficiency, and it lowers the operational costs of a process [48]. In cannabis processing, investigation into MW heating as a pre-treatment is scarce. Few approaches to MW-assisted extraction of cannabinoids are available. MW pre-treatments prior to distillation were performed by Fiorini et al. [49] for the enrichment of cannabidiol in the essential oil of industrial hemp inflorescence. They reported that MW pre-treatment had a significant effect (F13,27 = 28.229, *p* < 0.0001) in increasing the CBD level of cannabis inflorescences. According to their findings, a 3 min MW treatment with a power of 450 W and a 1 min MW treatment with a power of 900 W yielded 8.90% and 9.00% CBD, respectively, and the values were nearly two-fold above the untreated inflorescences that yielded 4.9% CBD. Overall, concerning different bioactive compounds, such as CBD, (*E*)-β-caryophyllene, and caryophyllene oxide of the cannabis inflorescence, MW treatment for 1 min with a power of 900 W was the most useful strategy [49]. MW-assisted extraction of phytochemicals from different plants, leaves and seeds is practiced all over the world. MW pre-treatments on rosemary leaves resulted in almost two times the phytochemical concentration during storage compared to the untreated leaves [50]. Phytocannabinoid extraction with the assistance of MW shows a significant improvement of the process output [51,52,53,54]. To prompt the drying process and to improve the polyphenol, cannabinoid, and terpene profile of cannabis, MW heating could be an ideal pre-treatment. However, optimum MW power and time should be comprehensively investigated.

##### Cold Plasma

**Figure 6:**
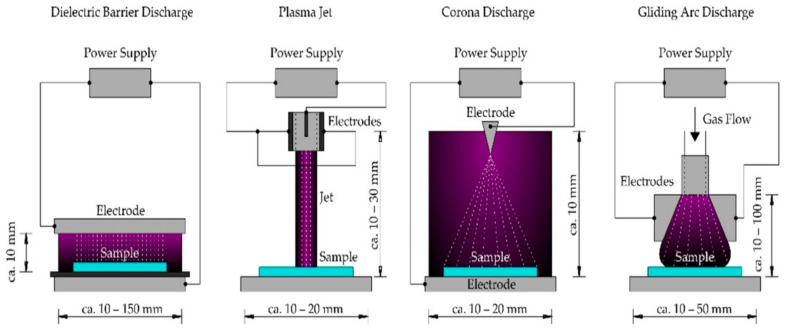
Schematic overview of cold plasma devices (adapted from Domonkos et al. [59] with permission).

Cold plasma (CP) is a new, non-thermal technique that may eradicate germs, inactivate enzymatic processes, enhance product quality, and assure product safety [55,56]. Plasmas are ionized gases that include a variety of electrons, ions, and neutral reactive species [57,58]. At atmospheric pressure and low temperature, a variety of gases (air, nitrogen, helium, and argon) and methods (corona discharge, dielectric barrier discharge, and plasma jet) are employed to create plasma [59]. Figure 6 represents different types of cold plasma devices. Plasma sources can be delivered to the object either directly (sample comes into direct connection with the plasma area) or indirectly (sample contacts the plasma or plasma-activated media) [60]. Cold plasma has been investigated for enhancement of the drying efficiency and quality of different foods, medicinal plants, and herbs, such as shiitake mushrooms [58], chili peppers [61], corn kernels [62], wolfberries [63], jujube slices [64], saffron [65], grapes [66], lemon verbena [67], coneflower leaves [68], and many others. All of these authors remarked that CP pre-treatment can significantly increase the drying efficiency and product quality concerning phytochemicals or microbial safety. In medicinal cannabis inflorescences, CP pre-treatment resulted in a decrease in fungal count. In medicinal cannabis inflorescences, both uninoculated and artificially infected by *B. cinerea*, cold plasma treatment for 10 min led to a 5-log-fold decrease in overall yeast and mold levels both in the control and the inflorescence of medicinal cannabis infected with the fungus [69]. Thus, the treatment of cannabis prior to drying, namely CP treatment in this case, may help to improve the shelf stability of dried cannabis with a shorter time required for drying. The time required for plasma treatment and the optimum distance between the plasma flume and inflorescence need to be comprehensively studied before industrial application of the technology for cannabis.

##### Pulsed Electric Field

**Figure 7:**
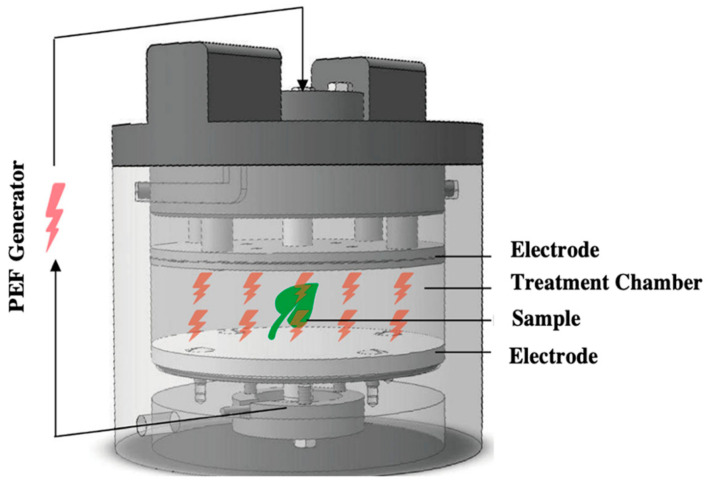
Schematic diagram of a typical PEF chamber (reproduced from Liu et al. [74] with permission).

Pulsed electric field (PEF) is a non-thermal approach that includes applying extremely short and high voltage pulses to a sample(s) positioned within electrodes, causing the development of new membrane pores, cellular membrane breakdown, and intracellular fluid release [70,71,72]. As this technique does not rely on heating, it may be used as a superior alternative to typical heat-transfer operations during the processing of biological materials. Although the mechanics of this technique is unknown, it is commonly referred to as electroporation or electropermeabilization. The term electroporation means either the formation of holes in the cellular membrane of the material or increase in the size of existing pores in the material, or a breakdown of the cell membrane continuity of the material [73]. Liu et al. [74] mentioned two important components of a typical batch PEF system: (a) a PEF generator and (b) a treatment chamber, as shown in Figure 7.

The drying rate, one of the important targeting properties of a drying operation, is improved by PEF as it changes the cellular structure of the plant materials. The treatment of apple tissues with 10 kV/cm and 50 pulses reduced the drying time up to 12% [72], while the application of an electric field strength of 1000 V/cm and 30 pulses with a pulse width of 120 ms resulted in the improvement of productivity per unit area and drying rate by 28.50% and 27.02%, respectively, as well as in the reduction of specific energy consumption and drying time by 20.46% and 22.50%, respectively [75]. Similar desired outcomes were also observed for carrots [72,76,77], potatoes [78], onions [79], Thai basil leaves [80], and red bell peppers [81]. In addition to drying, PEF also helps in the recovery of more bioactives and phytochemicals. Several studies are available on the PEF-assisted extraction of bioactive components from different foods and plant materials. When a plant cell is exposed to an external electromagnetic field, pores develop in the membrane, permitting the contents of the cell, such as phenolic components, to be left out [82]. The PEF-assisted extraction of polyphenol from fresh tea leaves at an electric field intensity of 1.00 kV/cm with 100 pulses of 100 s pulse length and 5 s pulse repetition resulted in an approximately two times higher extraction rate without significant alteration of the polyphenol profile [74]. For extracting intracellular components from microalgae [83], red beetroot pigment [84], anthocyanins from purple-fleshed potato [85], phenolic compounds from Merlot grapes [86], polyphenols from grape skin [87], and many more, PEF has shown beneficial results. In the case of cannabis, application of PEF for drying or extraction is rarely noticed. A study on the PEF-assisted extraction of oil and phenolics from cannabis with a PEF intensity of 3 kV/cm and a press speed of 20 rpm resulted in an increased oil extraction rate and the highest phenolic concentration (2036 ppm) [88]. PEF has also been used as a pre-treatment for ultrasonic extraction to improve the extraction yield of polyphenols from a defatted hemp seed cake [54]. Thus, PEF can potentially be employed to improve drying efficiency due to decreased drying times as well as the retention of important phytoconstituents of cannabis. Further detailed studies on this matter are suggested.

##### Ultrasound

Sound is a mechanical wave that is created by the vibration of materials. Mechanical waves have a frequency range of less than 16 Hz to more than 1 GHz and are grouped into four different categories based on the frequency of waves. If the mechanical waves range between 1 to 16 Hz, they are infrasound; acoustic or audible sounds range between 16 to 20 kHz; ultrasound has a frequency range of 20 kHz to 1 GHz; and hypersound has a frequency of 1 GHz and above [89]. Ultrasounds are created using a variety of processes, including mechanical (aero- and hydrodynamics), thermal (electric discharge), optical (high-power laser impulse), and reversible electric and magnetic technologies (piezoelectric, electrostriction, and magnetostriction). Recently, the application of ultrasound for the processing and preservation of food and biomaterials has gained significant interest from scientists. Few applications of ultrasound in food processing include thermal processing (sterilization and pasteurization), freezing and thawing, emulsification, viscosity modification, polymerization, extraction of different compounds, tissue interruptions, aggregate dispersion, crystallization, etc. [89]. For many years, ultrasonically aided drying has piqued curiosity. This technology is being applied as a pre-treatment to improve drying kinetics as well as to minimize the energy cost associated with drying [90,91,92]. Ultrasonic waves in a solid cause a quick succession of alternate contractions and expansions of the substance through which they travel [93,94]. This alternating stress forms tiny channels, aiding in moisture transfer. Furthermore, highly intense acoustic waves cause the detonation of strongly bound water within the solid material, assisting in its evaporation [93]. There is a plethora of research on ultrasound-assisted drying to enhance drying parameters and product quality. The ultrasound-assisted vacuum drying (USVD) of red peppers resulted in a 25% reduction in drying time and an 89% enhancement of the effective moisture diffusivity without a substantial loss of bioactive components [95]. In the case of carrot slices, USVD reduced drying time by 41–53% and helped in retaining more beta-carotene, ascorbic acid, color, and texture [91]. For papaya, an enhanced drying rate with the lowest loss of ascorbic acid (41.3%) was observed when the samples were dried with the aid of ultrasound and vacuum [96]. In the case of apple tissue, ultrasonic treatment resulted in a lower drying time (31%) and density (6–20%) as well as higher shrinkage (9–11%) and porosity (9–14%) compared to the untreated apple tissue [92]. The addition of solvents like ethanol to ultrasound treatments constitutes another technique as it has resulted in the greatest reduction in both the drying time (59%) and energy consumption (44%) for pumpkin [97]. Ultrasound also improved the drying time for other foods, such as: (a) pineapple with a drying time reduction of 8% [98], (b) persimmon [93] and raspberries with a drying time reduction of 54–64% [99], and (c) purple-fleshed sweet potato with a reduction in the drying process energy consumption of up to 34.60% [74]. Several studies are also available on the extraction of bioactive compounds with the assistance of ultrasound from different sources, such as wild ginger [100], pomegranate peel [101], myrtle (*Myrtus communis* L.) [102], green tea [103], tobacco waste [104], etc. In the case of cannabis, this technology can play a very important role, especially for the reduction of drying time and energy consumption as well as increasing the cannabinoid profile. Ultrasound as a pre-treatment for the drying process has not yet been investigated for cannabis, although few studies were performed to improve the extraction of cannabinoids. Ultrasound-assisted extraction (UAE) was used to collect volatile chemicals from cannabis inflorescences. Ultrasonic treatment of *Cannabis sativa* L. cultivars for less than 5 min resulted in higher terpene concentrations than maceration, while ultrasonic treatment for more than 5 min resulted in higher THC concentrations [105]. Furthermore, Agarwal et al. [106] concluded that ultrasound considerably improved the extraction of cannabinoids present in cannabis. Ultrasound also can increase the recovery of oil from cannabis [107,108]. Considering the above outcomes from the literature, ultrasound can be an important treatment for the cannabis industry to enhance the drying process and extraction of cannabinoids. Thus, a feasibility study of this technique along with optimization in case of cannabis is highly recommended.

##### Irradiation

Exposing materials to ionizing radiation is known as irradiation. Ionizing radiation can remove electrons from atoms and molecules, causing them to become ions. Ionizing radiation is made up of high-energy charged particles, such as electrons, and photons, such as X-rays and γ-rays. However, due to limited penetrating power (alpha particles) or because they create radioactivity in the irradiated substance, all types of ionizing radiations are not used for irradiation; only three types of ionizing radiations (X-rays, γ-rays, and electron beams) are used for irradiation [109]. Irradiation treatment for the decontamination of cannabis is very common. More than 80% of licensed growers of cannabis in Canada are using irradiation treatment [110]. Medical cannabis treated with a ≥10 kGy irradiation dose from Cobalt-60 as radiation source resulted in a significant decontamination of the cannabis without largely affecting the phytocannabinoids [111]. However, irradiation has a significant use for enhancing the drying rate of different foods either using gamma irradiation or electron beam irradiation. Most of the research has been carried out using 60-Co γ-rays with a dose of up to 12.0 kGy. Irradiation pre-treatments resulted in a higher drying rate for potato and apple [112], carrot, potato and beetroot [113], and tofu protein [114]. A few researchers have performed irradiation as a pre-treatment for cannabis [110,111,115]. The level of irradiation, optimization, and its effect on phytocannabinoids are yet to be investigated.

### 3.3. Curing

Curing in agricultural industries often refers to the maturation of the chemical profile of a biological material over long periods of time to obtain optimum quality and flavor. This usually involves unique iterations of methods [104,116,117,118,119,120]. For the cannabis industry, curing involves essentially marking the end of a drying process and the beginning of the storage process, where the cannabis inflorescence is being maintained at an ideal moisture content and water activity. It is an important step to maintain the quality of cannabis. Interactions between the storage, method of curing, water activity, potency, chemical profile, and maturity of cannabis in this postharvest step are still not widely investigated. Color and texture changes of tobacco are determinants for curing [121] and a similar method to detect the more subtle changes in color, aroma, and texture in cannabis curing might be worth looking into.

For cannabis, this process includes keeping the dried inflorescence in a closed container for a certain period with a specific temperature and humidity. When a plant is cut, enzymes and aerobic bacteria start their activity to break down the extra sugars and starches created by chlorophyll breakdown. When smoking cannabis that has not been properly cured, the presence of these residual sugars and minerals causes a burning sensation in the throat. Before the breakdown of the remaining sugars, carbohydrates, and nutrients, the plant is forced to utilize these compounds in flavored items during the curing process. Thus, this resting period introduces less odor and sensation of throat burning when smoking or inhaling. It can also result in an increase in the level of THC and CBN by completing the decarboxylation process, as well as extending the storability by limiting fungal growth [29]. According to Jin et al. [28], maintaining a temperature of 18 °C and an RH of 60% for a period of 2 weeks, with the opening of the lid after 6 h, is the best curing condition. Although curing is one of the most significant postharvest operations, it has been overlooked and is not investigated properly. The time, temperature, humidity, and storage container during curing may be varied and should be investigated more to get the best product for storage and marketing.

### 3.4. Packaging and Storage

The cannabis inflorescence, with its high initial moisture content at harvest ranging from 75–78% (wet basis, wb), is considered to be a highly perishable material before drying. A common problem arising in the methods for the drying of cannabis is the inadequate control and prediction methods used to dry and store cannabis, resulting in the formation of mold [10,64,122].

Important factors to consider for quality, in terms of the packaging material, are water permeability to prevent moisture inconsistencies and rigidity in preventing mechanical injury. The ability to predict the movement of moisture throughout a biological material such as cannabis is critical to further improve quality problems in the industry [10]. Diffusion calculations through a membrane are similar to the calculations that use Fick’s law as outlined by Geankoplis [25] and Srikiatden and Roberts [33]. Permeabilities for packaging materials are often widely available for consumers to check. A holistic approach considering the diffusion coefficient, sorption isotherms, and the permeability of packaging materials allows producers to make critical and efficient decisions to further improve their production process.

Common packaging materials used in the cannabis industry are Mylar bags, PET (polyethylene terephthalate) and/or metal cases and tubes, and glass jars, as found in online catalogs [123,124]. Water vapor permeability, or the measure of how fluid easily flows through a substance [125], plays a role in achieving the optimum quality measures needed for the industry. Mylar bags have a permeability ranging from 0.02 to 1.18 cm^3^ mm/m^2^ d atm [126]. This means that dried cannabis inflorescence being stored in bags made of Mylar gradually lose moisture as cannabis is stored in inventory for months and even years.

Storage conditions would also contribute to the degradation of cannabis and the cannabinoids and volatile compounds present in them. Trofin et al. [127,128,129] have extensively investigated the stability of various cannabis products, focusing on major cannabinoids via kinetic models and in-vitro studies, and have found that the storage of cannabis at 22 °C with the presence of light is highly detrimental to cannabis compared to samples stored in darkness at 4 °C. These results are also significantly similar to studies by Taschwer and Schmid [130] and Chen et al. [34], where the total amount of major cannabinoids present in cannabis, such as THC and CBD, decreased as the storage and drying temperatures increased. Temperature control during storage therefore plays an important role in best quality practices in maintaining both moisture content and cannabinoid concentration in dried cannabis inflorescence.

## 4. Effect of Postharvest Processing on Phytoconstituents of Cannabis

The main thermal treatment for cannabis is drying. In addition to that, few other treatments have been investigated by researchers to determine their effects on product quality. Though research in this area is hardly available in the literature, this section will summarize the recent output of different studies focused on temperature dependence of cannabinoids during drying. Table 1 represents the effect of postharvest treatments on the cannabinoid profile of cannabis. From the overall findings, it can be remarked that the cannabinoid content varies with the drying parameters, drying methods, storage temperature, extraction solvents, and other treatments.

Chen et al. [34] studied the effect of different drying techniques, e.g., freeze drying (FD); ambient air drying at 22.3 °C and 37.9%; hot air (HA) drying at 40, 50, 60, 70, and 90 °C (with air flow rate of 1.4 m/s); sequential infrared and hot air (SIRHA) drying (dried for either 1 or 2 min by IR and sequentially dried by HA drying at 60 °C or 40 °C for 1 or 2 min, respectively) on three different varieties (Pipeline, Maverick, and Queen Dream CBD) of *Cannabis sativa* inflorescences. The total THC content of the sample was below 0.3% while the CBD content varied due to the variation of treatments. FD resulted in 7.76 ± 0.03 to 13.93 ± 0.03 g total CBD/100 g of dry matter. HA drying resulted in a higher CBDA conversion rate or more decarboxylation than that of FD. Application of IR drying improved the drying rate but it was found that it can cause the loss of CBD [34]. Challa [131] worked on *Cannabis sativa* buds using FD, HA drying (25, 32, 40, 50, 60, and 75 °C with an air flow rate of 1 ± 0.1 m/s and 45–60% RH), and non-isothermal drying (initially dried at 40 °C and increased to 70 °C at 45% or 25% moisture content (MC); again, dried initially at 40°C and increased to 60 °C at 45% or 25% MC) to determine the effect of the different drying methods on the cannabinoid profile. They obtained 1.91% of total CBD in fresh cannabis buds, which gradually increased with the increasing drying temperature of HA drying; however, they did not observe any significant difference (*p* > 0.05) in terms of total CBD content due to the changes in the drying conditions. They observed that decarboxylation did not largely affect the CBD:CBDA ratio, although the total CBD content varied due to drying methods and conditions. The maximum total CBD was obtained from non-isothermal drying starting at 40 °C but increased to 70 °C when the MC decreased to 25% [131].

A study on *Cannabis sativa* inflorescences collected from three different portions of the stem (upper, middle, and lower) was conducted by Namdar et al. [132] to explore the effect of three different drying methods: stream of nitrogen, vacuum, and rotary evaporator, along with three different extraction solvents, such as ethanol, n-hexane, and a hexane + ethanol solution. They reported that the upper portion of cannabis plants contained more cannabinoids, followed by middle and lower portion. They also reported that the extraction capacity of ethanol is higher than n-hexane and the hexane + ethanol solution. Thus, the extraction techniques and solvents have a significant role on the quantity of the cannabinoids. Valizadehderakhshan [133] reviewed the modern extraction methods along with possible pre-treatment practices to enhance the extraction efficiency for large-scale processing. These techniques may include microwave-assisted extraction, supercritical fluid extraction, maceration, etc. Thermal treatments other than drying can also alter the cannabinoid profile of cannabis. The effect of sterilization at a temperature of 62.5–70 °C for 10–20 s was studied by Jerushalmi et al. [134] and revealed that sterilization can reduce the contained phytocannabinoids but not by more than 20%. In situ decarboxylation of *Cannabis sativa* was analyzed by Nuapia et al. [135] at a temperature of 80–150 °C for 5–60 min using pressurized hot water extraction techniques. The findings revealed that decarboxylation increased with the temperature and time, but after 150 °C the decarboxylation rate decreased with time. Microwave heating on size reduced cannabis resulted in almost 1.8 times higher CBD content than the untreated cannabis sample, which reveals that microwave pre-treatment can increase the cannabinoid content [49]. Irradiation can also help in increasing the active compounds of cannabis. Work has been conducted on four different cultivars of *Cannabis sativa* sp. *indica* to determine the effect of irradiation on THC levels and it was reported that THC significantly increased due to irradiation treatment [110]. Storage analysis revealed that the higher the storage temperature, the higher was the degradation of phytocannabinoids and it was suggested that a 4 °C temperature could be the optimum for storage of cannabis along with olive oil [136,137]. Considering these few pieces of literature, it can be summarized that decarboxylation of cannabinoids to their neutral forms depends on thermal treatments applied during post-harvest processing. However, more investigations have yet to be conducted to determine the optimum drying methods and conditions, pre-treatments, extraction methods and time, storage, and many others.

**Table 1:** The effect of postharvest treatments on the cannabinoid profile of cannabis.

Sample	Moisture Level (wb)	Experimental Details	Findings	Source
Inflorescence and leaves of hemp (three varieties: Pipeline, Maverick and Queen Dream CBD)	Initial: 75–78%, Final: 9–13%	Freeze drying, Ambient drying, Hot air drying, and Sequential infrared and hot air (SIRHA) drying	Increased drying rate with the increasing of temperature Enhanced decarboxylation of CBDA from 0.2% to 14.1% and reduced terpene retention from 82.1% to 29.9% when the drying temperature increased from ambient to 90 °C SIRHA drying resulted in significant losses of CBD and terpene up to 16.2% and 72.3%, respectively	[34]
Hemp buds	Initial: 65%, Final: 10%	Freeze drying, Hot air drying, Non-isothermal (stepwise) drying	Phytocannabinoids and drying time were significantly affected by drying techniques and conditions Drying temperatures over 40 °C significantly reduced terpene concentration, but no effect on the CBD level Decarboxylation (CBDA to CBD) increased with temperature and maximum at 70 °C Highest amount of CBD in non-isothermal drying samples	[131]
Inflorescences of medicinal cannabis (*Cannabis sativa)*	Not mentioned	Steam sterilization for 10s at 62.5°C, 15 s at 65 °C and 20 s at 70 °C	Steaming caused minor reduction of terpenes and CBD (<20%)	[134]
Powder of *Cannabis sativa* seeds	Initial: Not mentioned, Final: 3.5–5.1%	In situ decarboxylation using pressurized hot water extraction technique at temperature (80 to 150 °C) for 5 to 60 min	Decarboxylation to CBD and THC increased with time and temperature but THC decreased with time at 150 °C Optimal decarboxylation time and temperature were 42.2 min and 149.9 °C, respectively	[135]
Inflorescences of hemp cv Felina 32	Not mentioned	Steam distillation (SD) or hydro distillation (HD) of fresh sample; HD of ambiently dried inflorescences; HD of blended and powdered inflorescences; HD of powdered and heated (120 °C for 1, 3, or 6 min) inflorescences; HD of powdered and microwaved (900 and 450 W) inflorescences	HD recovered higher cannabinoids over SD Pretreatments and drying triggered the cannabinoid profile; Microwave heating resulted almost double CBD MW heating for 1 min at 900 W was the most effective approach for the best quality products	[49]
*Cannabis sativa* inflorescence collected from upper, middle, and lower portion of stem	Not mentioned	Solvent extraction (ethanol, n-Hexane, mixture of hexane and ethanol (7:3, *v*:*v*)) of undried and dried (using gentle stream of nitrogen, vacuum dryer and rotary evaporator)	Extraction ability of solvents: ethanol > n-hexane > hexane and ethanol solution Cannabinoid and terpene quantity was influenced by drying methods and declined as the sampled flower moved from upper to middle to lower	[132]
Inflorescences of *Cannabis sativa* (cultivars: Pink, RBS, RMS, and GSC)	Not mentioned	Dried and irradiated with 5 kGy emitted from a 10 MeV accelerator	Irradiation affected the THC and terpenes Except for RMS, three of the four cultivars examined showed a significant rise in THC levels Three of the four extracts studied had their anti-cancer capabilities modified after being irradiated	[110]
Two *Cannabis sativa* strains combined together, sieved through a 355-mm sieve, and homogenized (one strain contained primarily THCA/THC and the other contained CBDA/CBD).	Not mentioned	Stored in 66-L microbiological incubators with ±0.2 °C consistency for up to 52 weeks at different temperature (20 °C, +4 °C, +20 °C, +32 °C, +37 °C, and +40 °C).	Cannabinoids followed 1st order reaction kinetics during storage and also affected by temperature Lowering temperature by 5 °C doubled the shelf life of 85% cannabinoids.	[136]
Inflorescences of medicinal cannabis (one is ∆9-THC-rich and another is CBD rich)	Initial: Not mentioned Final: 9 ± 0.3%	Samples stored in the dark condition for 12 months at 4 distinct temperatures (−80, −30 °C, 4 °C, and 25 °C) and in 2 physical forms (whole or ground).	Storage at 25 °C affected mostly on phytocannabinoid concentrations over time Dissolving the whole inflorescences or extracts in olive oil and stored at 4 °C was the ideal postharvest storage conditions	[137]
Inflorescences and leaves of hemp	Initial: 65.7% Final: up to constant moisture ratio	Convective drying at constant (40, 50 and 60 °C) and time varying temperature rise (1.5, 2.5 and 4 °C/h) at temperature in the 40–60 °C range	Time varying temperature drying resulted in significantly higher CBD mean value for inflorescences (+46.7%) and leaves (+65.3%), but not significant for THC level	[138]

CBD = Cannabidiol; THC = tetrahydrocannabinol; THCA = tetrahydrocannabinolic acid, CBDA = cannabidiolic acid; wb = wet weight basis; SIRHA = sequential infrared and hot air; SD = steam distillation; HD = hydro distillation; MW = microwave.

## 5. Summary and Future Perspectives

The ongoing demand for cannabis due to its medical and recreational uses, and its consequent legalization by the governments of several countries, enhances the development of the cannabis industry. It has come to the attention of scientists and engineers to understand its chemistry and uses. Harvested cannabis goes through different operations, such as trimming, drying, curing, storage, and packaging, in different forms of the final product. Previous work on postharvest treatments indicates that slow drying is the most practiced drying method in the cannabis industry, thought it has several drawbacks like long drying time, chances of microbial contamination, and undefined equilibrium moisture level. Researchers are trying to find suitable postharvest operations for cannabis. Still, a complete guideline for the postharvest processing of cannabis is lacking. This review suggests practical research on finding the best suitable drying method for cannabis along with optimum conditions, equilibrium moisture content of the final product, suitable pre-treatments, curing conditions, and packaging materials. To improve the drying profile and product quality, different modern technologies such as microwave, cold plasma, pulse electric field, radio frequency, etc., can be applied. The determination of a suitable sorption isotherm model(s) and the optimum equilibrium moisture content considering cannabis’ physical characteristics is also important and should be considered for proper drying and storage management.

## Data Availability

The authors confirm that the data supporting this study are available within the article.
